# Supplementary material for: Cytoreductive surgery and hyperthermic intrathoracic chemotherapy in thymic epithelial tumors with pleural spread or recurrence: a prospective, single-arm, phase II study
Source: Nat Commun. 2025 Jun 4;16:5175. doi: 10.1038/s41467-025-60386-0 (PMC12137697; doi:10.1038/s41467-025-60386-0)
Supplement: Supplementary file 1 — Supplementary Information [file 41467_2025_60386_MOESM1_ESM.pdf]

Supplementary Information of Cytoreductive Surgery and Hyperthermic Intrathoracic Chemotherapy in Thymic Epithelial Tumors with Pleural Spread or Recurrence: A Prospective, Single-Arm, Phase II Study

Supplementary Note 1: supplementary tables 1-2 and supplementary figures 1-10.

Supplementary Note 2: the full study protocol.

Supplementary Note 1

Supplement Table1. History of treatment in the all enrolled patients.

Variables	N (%)
DNT (N=12)	
Preoperative treatment	
None	5 (41.7)
Radiotherapy	1 (8.3)
Chemotherapy	3 (25.0)
Chemoradiotherapy	3 (25.0)
TPR (N=33)	
Adjuvant therapy after first surgery	
None	10 (30.3)
Radiotherapy	11 (33.3)
Chemotherapy	2 (6.1)
Chemoradiotherapy	10 (30.3)
Post-recurrence treatment	
None	25 (75.8)
Radiotherapy	4 (12.1)
Chemotherapy	1 (3.0)
Chemoradiotherapy	3 (9.1)

DNT, de novo Masaoka stage IVA TETs; TPR, TETs with pleural recurrence.

Supplement Table 2. Univariable and multivariable analyses of PFS.

Variables	Univariable analyses		Multivariable analyses	
	HR (95% CI)	P Value	HR (95% CI)	P Value
Age (≥60 years vs. <60 years)	0.62 (0.10-3.7)	0.603	-	-
Sex (male vs. female)	0.30 (0.03-3.01)	0.309	-	-
CCI (≥ 4 vs. < 4)	0.72 (0.41-1.26)	0.247	0.79 (0.46-1.33)	0.368
Myasthenia gravis (yes vs. no)	0.69 (0.08-6.22)	0.741	-	-
Histological type (TC vs. thymoma)	0.87 (0.09-8.13)	0.906	-	-
Type of pleural metastasis (TPR vs. DNP)	3.78 (0.40-36.05)	0.248	0 (0-Inf)	>0.999
pT stage (pT+ vs. pT0)#	0.15 (0.01-1.50)	0.105	0 (0-Inf)	>0.999
pN stage (pN+ vs. pN0)#	0.85 (0.09-7.82)	0.884	-	-
Neoadjuvant therapy (yes vs. no)	1.25 (0.19-8.16)	0.818	-	-
Resection (R2 vs. R0+R1)	0.25 (0.03-2.34)	0.224	0.23 (0.02-2.37)	0.219
PTI (Continuous variables)	1.72 (1.10-2.69)	0.017	2.76 (0.85-8.96)	0.092

CCI, Charlson Comorbidity Index; TC, thymic carcinoma; pT+, postoperative pathological T stage positive; pN+, postoperative pathological N stage positive; DNT, de novo Masaoka stage IVA TETs; TPR, TETs with pleural recurrence; R0, complete cytoreductive surgery without residual visible disease; R1, optimal cytoreductive surgery with residual tumors measuring no more than 10 mm; R2, incomplete cytoreductive surgery with residual lesions measuring > 10 mm in diameter; PTI, pleural tumor index; HR, hazard ratio.

thymic malignancies were graded by AJCC/UICC/IASLC/ITMIG TNM stage (9th edition).

Supplement Figure 1. Inflow and outflow temperature control during hyperthermic intrathoracic chemotherapy.

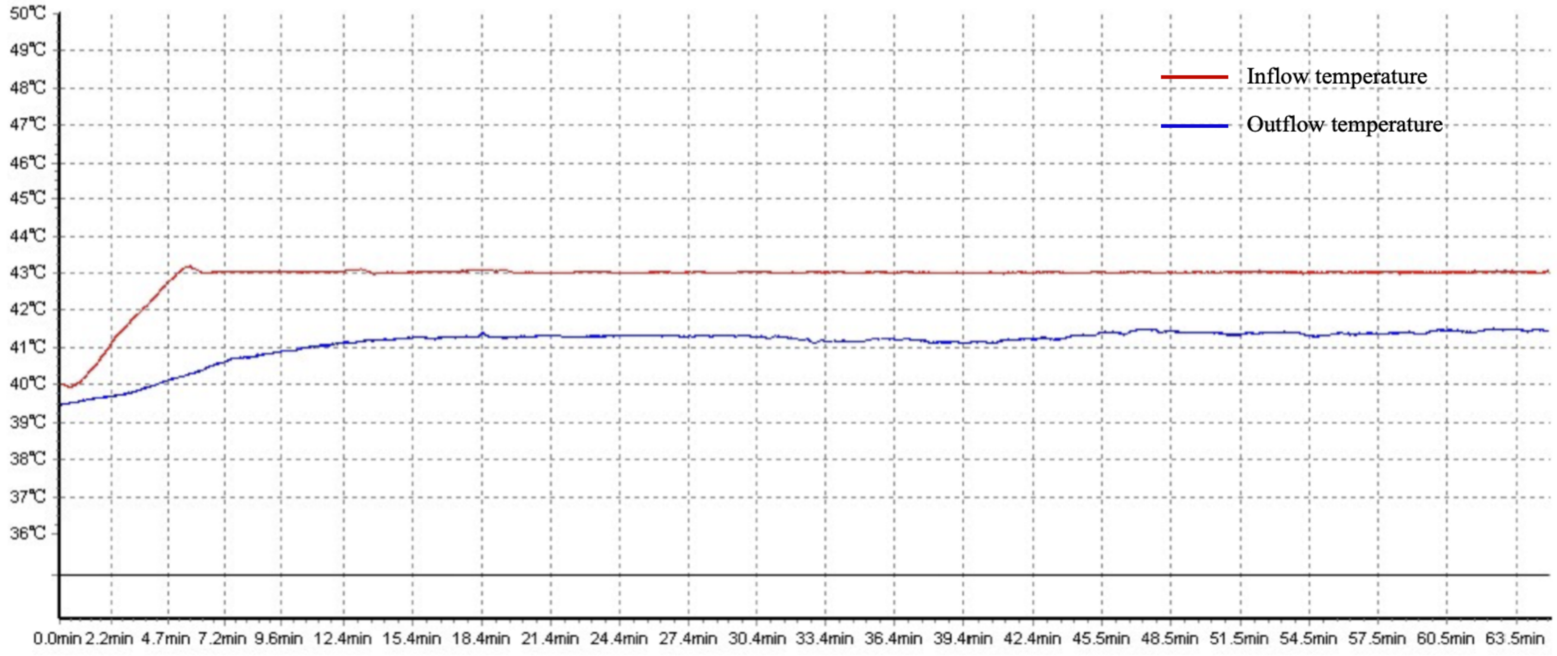

During the 60-minute hyperthermic intrathoracic chemotherapy, the inflow and outflow temperatures were stable.

Supplement Figure 2. The screening flowchart in this study.

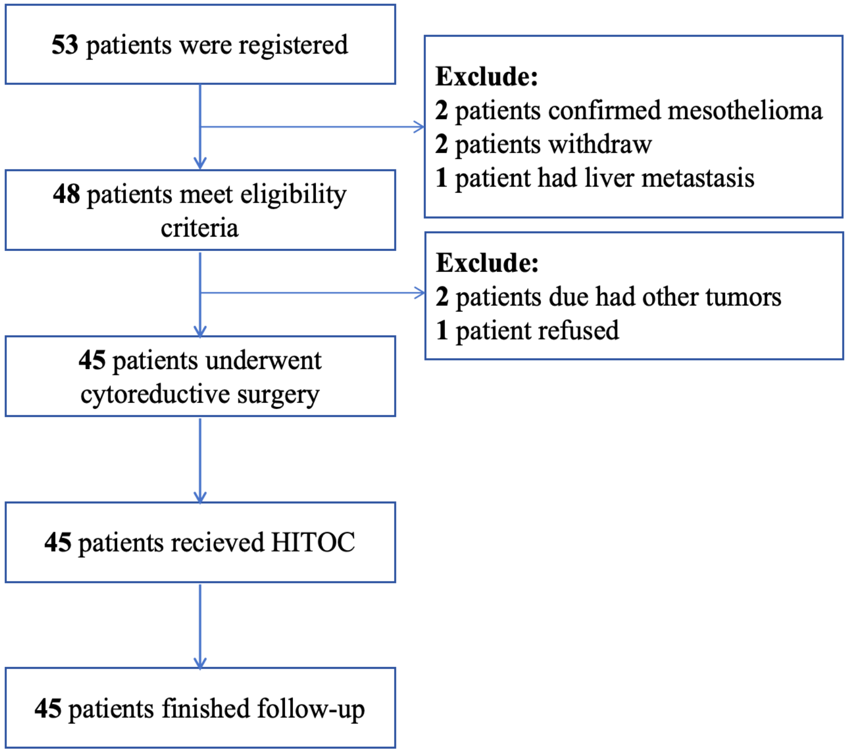

Supplement Figure 3. Typical surgical images during pleurectomy/decortication and extended pleurectomy/ decortication.

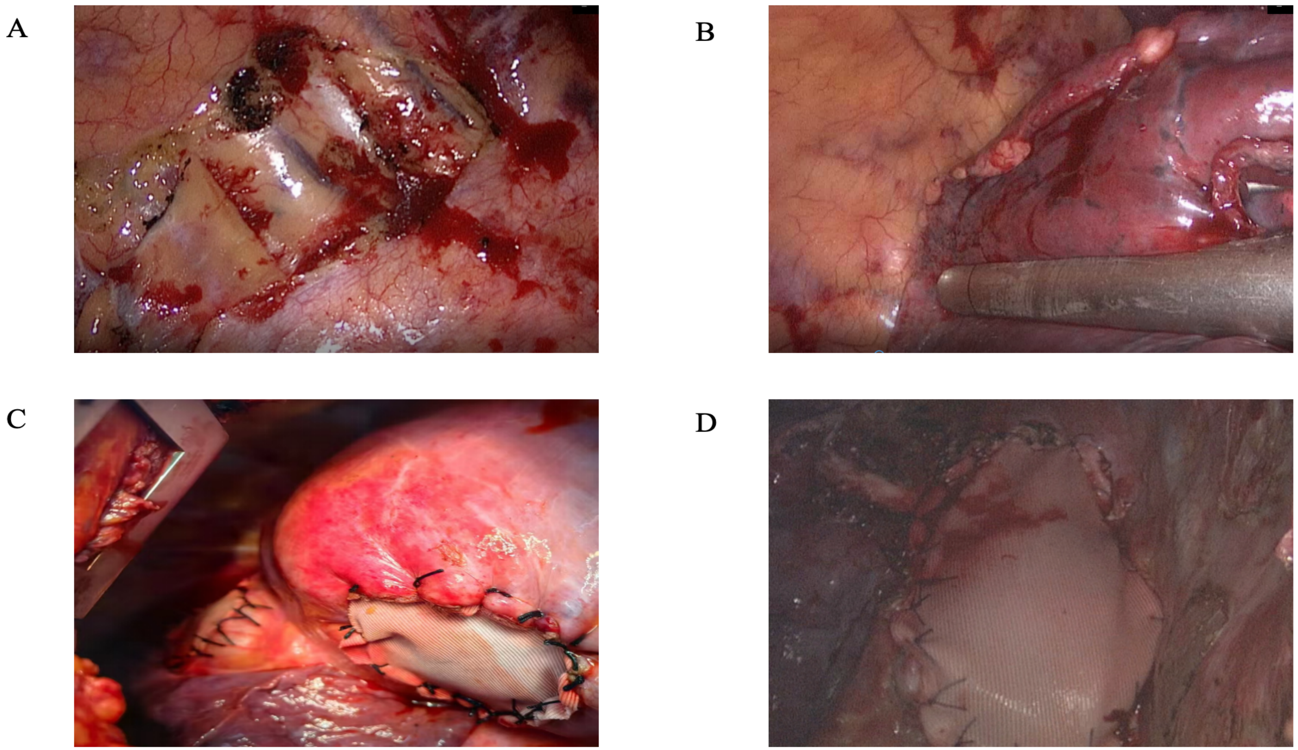

(A) resection of pleural metastatic nodules, (B) lung wedge resection, (C) partial diaphragmatic resection and repair, and (D) partial pericardiectomy and repair.

Supplement Figure 4. A schematic diagram of the removal of invaded tissues.

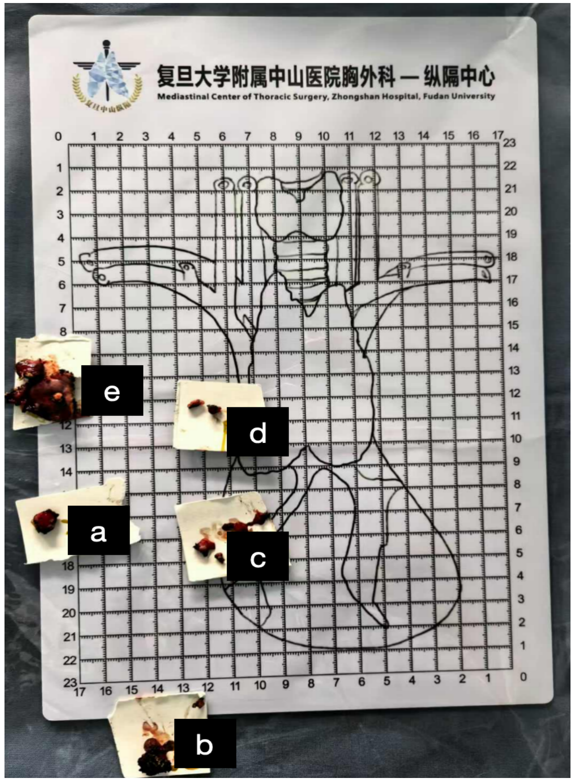

The resected tissues included (a) pleural metastatic nodules [Zone I], (b) partial diaphragm [Zone II], (c) partial pericardium [Zone III], (d) pleural metastatic nodules [Zone III], and (e) partial lung tissue [Zone V].

Supplement Figure 5. Baseline and posttreatment pain assessments of participating patients.

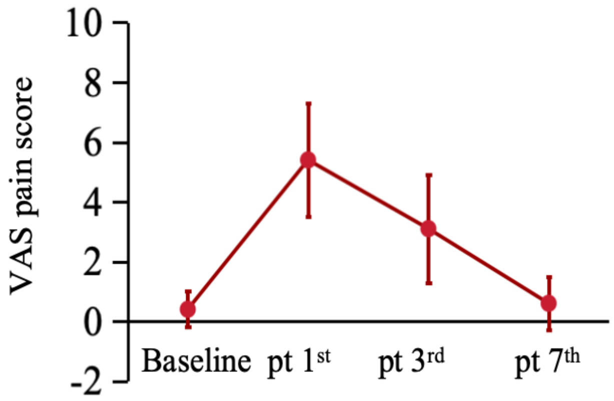

Time Point	VAS pain score, mean ± SD	P value (vs. Baseline)
Baseline	0.4 ± 0.6	-
pt 1st	5.4 ± 1.9	<0.001
pt 3rd	3.1 ± 1.8	<0.001
pt 7th	0.6 ± 0.9	0.220

N=45; pt 1st, posttreatment 1st day; pt 3rd, posttreatment 3rd day.

Supplement Figure 6. Baseline and posttreatment QoL assessments of participating patients.

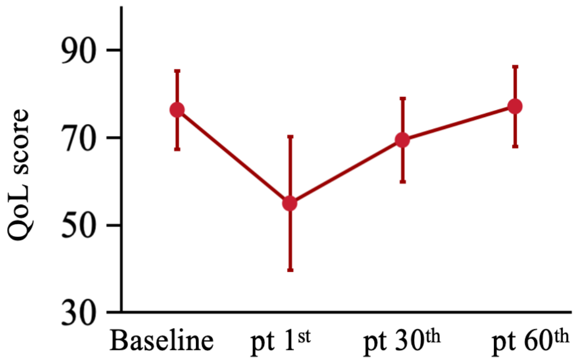

Time Point	QoL score, mean ± SD	P value (vs. Baseline)
Baseline	76.3 ± 8.9	-
pt 1st	54.9 ± 15.3	<0.001
pt 30th	69.4 ± 9.5	0.001
pt 60th	77.1 ± 9.2	0.318

N=45; pt 1st, posttreatment 1st day; pt 30th, posttreatment 30th day; pt 60th, posttreatment 60th day

Supplement Figure 7. MGFA post-intervention status among participating patients after S-HITOC.

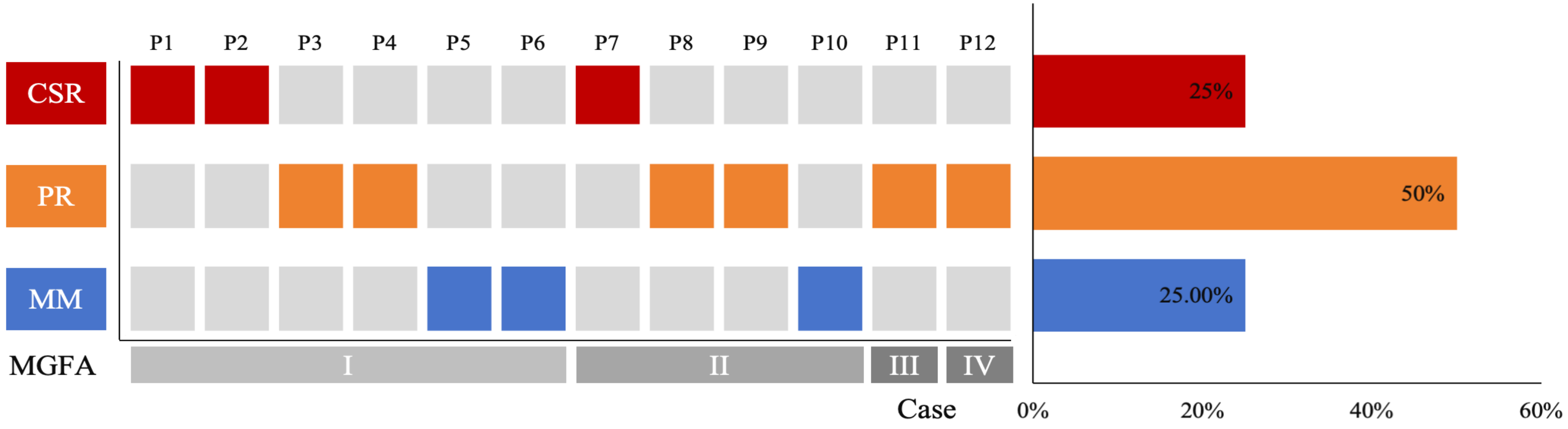

N=12; CSR, complete stable remission; PR, pharmacological remission; MM, minimal manifestations; MGFA, Myasthenia Gravis Foundation of America.

Supplement Figure 8. Mean glucocorticoid usage in patients with MG before and after (at the latest visit) S-HITOC.

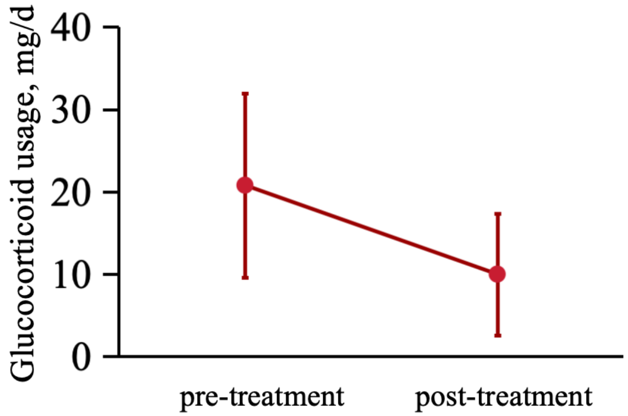

Time Point	Glucocorticoid usage, mg/d, mean ± SD	P value (post vs. pre)
pre-treatment	20.8 ± 11.2	0.011
post-treatment	10.0 ± 7.4	

N=12; post, posttreatment; pre, pretreatment.

Supplement Figure 9. Survival difference of patients in two groups with two pathologic types of thymic epithelial tumors.

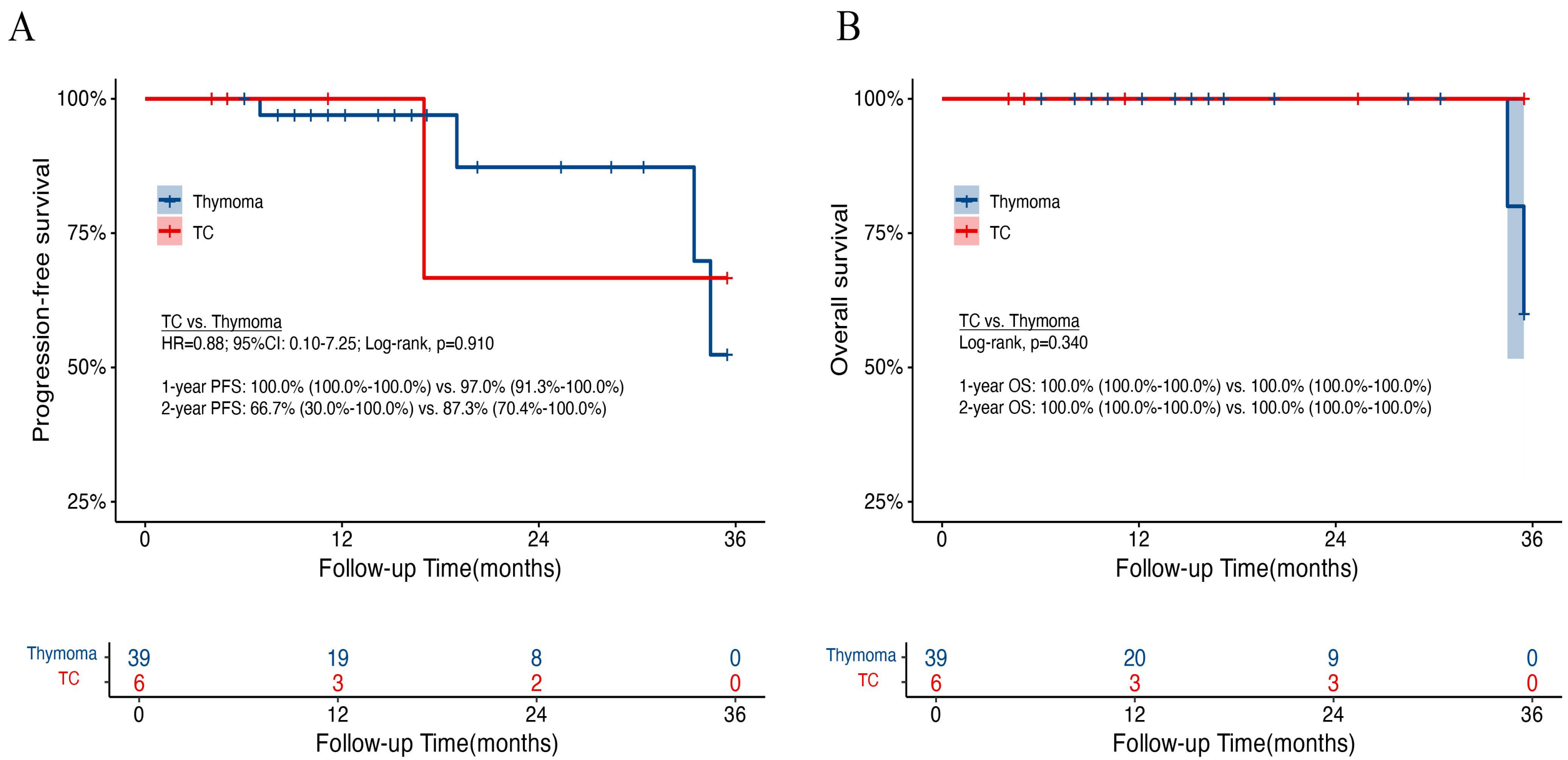

(A), Progression-free survival [N=45, N (Thymoma)=39, N (TC)=6]; (B), Overall Survival [N=45, N (Thymoma)=39, N (TC)=6]. TC, thymic carcinoma; PFS, Progression-free survival; OS,Overall Survival.

Supplement Figure 10. Progression-free survival difference between the patients with or without preoperative treatment.

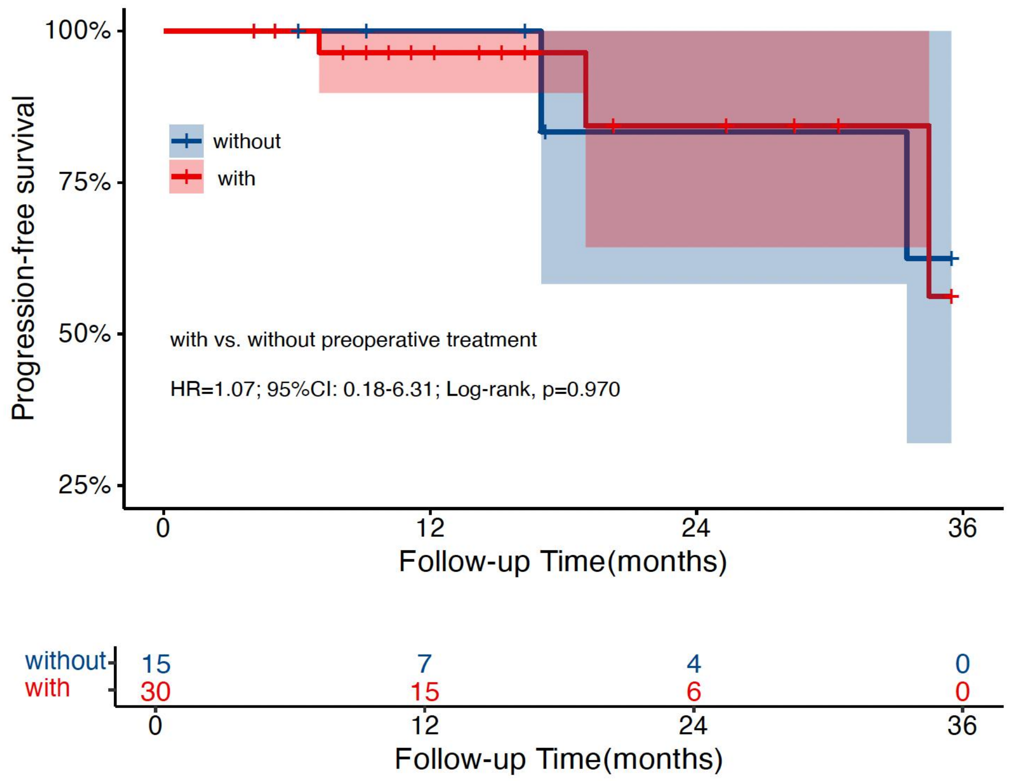

[N=45, N (with preoperative treatment)=30, N (without preoperative treatment)=15].

Supplementary Note 2：

Trial Protocol and Statistical Analysis Plan

Version (Final)　2021

Trial Title: Short-term Outcomes of Cytoreductive Surgery and Hyperthermic Intrathoracic Chemotherapy for Thymic Epithelial Tumors with Pleural Spread or Recurrence: A Prospective, Single-Arm, Phase II Study

Short title: S-HITOC for TETs with Pleural Spread or Recurrence

ClinicalTrials.gov Identifier: NCT05446935

Principle Investigator (PI)

Prof. Jianyong Ding

Add: Department of Thoracic Surgery, Zhongshan Hospital, Fudan University, Fenglin Road 180, Shanghai 200032, China.

Phone Number: 086-18616881268

Email: ding.jianyong@zs-hospital.sh.cn

Confidentiality Statement

The information contained in this clinical protocol is only available to the investigators, the Ethics Committee, and relevant agencies for review. Without an approval from the principal investigator (PI), any information shall not be informed to the third party irrelevant with this study.

1. Background and rationale

Thymic epithelial tumors (TETs), including thymoma and thymic carcinomas, are the most common anterior mediastinal tumors (1). An aggressive surgical approach is considered the mainstay of therapy for TETs, except for clinically non-resectable tumors or those with distant metastasis. TETs with pleural spread or recurrence are defined pathologically as Masaoka-Koga and Tumor-Node-Metastasis (TNM) stage IVa. TETs are locoregional and considered potentially resectable. However, the five-year survival rates of patients with TETs with pleural spread or recurrence have been reported to range from 33% to 46% in different studies and are significantly lower than those of patients with TETs without pleural spread or recurrence (2). Thus, it has been suggested that a multimodal treatment approach should be adopted to treat advanced-stage patients.

Currently, there is no standard approach for treating TETs with pleural spread or recurrence. However, there are several empirical therapeutic methods for treating patients with TETs with pleural spread or recurrence, including surgery combined with chemotherapy and/or radiotherapy. Multimodality treatments using neoadjuvant chemotherapy/radiotherapy followed by surgery or resection followed by adjuvant therapy have produced highly improved oncological outcomes and promising results in the treatment of patients with advanced diseases (3). However, studies focusing on a chemotherapy-based regimen for advanced TETs have indicated that the response rates were relatively heterogeneous, and ranged from 20% to 100% (4).

Cytoreductive surgery followed by hyperthermic intrathoracic chemotherapy (S-HITHOC) is an emerging technique that might improve the progression-free survival (PFS) and overall survival (OS) of patients with TETs with pleural involvement. In recent decades, several studies have reported that S-HITHOC can achieve the local excision of recurrent cancer and improve the control of local disease with satisfactory safety and efficacy **(Table 1) (**5-12).

Theoretically, hyperthermic intrathoracic chemotherapy (HITHOC) has the advantage of directly exposing the tumor to a locally higher concentration of the antineoplastic agent with fewer systemic adverse events. Further, the combination of hyperthermia with chemotherapeutic agents may have an additive effect on pleural malignancy by increasing the effectiveness, the penetration

depth, and the activation of apoptosis. To our knowledge, few studies have compared the results of surgery alone with those of S-HITHOC in the treatment of TETs with pleural spread or recurrence.

Table 1. Study overview.

Study	Patients	Chemotherapeutic agents	Duration	Temperature
Refaely et al. (5)	N = 15	Cisplatin (100 mg/m^2 BSA)	60 min	42°C
De Bree et al. (6)	N = 3	Cisplatin (80 mg/m^2 BSA); doxorubicin (15–30 mg/m^2 BSA)	90 min	40–41°C
Ried et al. (7)	N = 8	Cisplatin (100–150 mg/m^2 BSA)	60 min	42°C
Yellin et al. (8)	N = 35	Cisplatin (100 mg/m^2 BSA); doxorubicin (50–60 mg)	60 min	43°C
Yu et al. (9)	N = 4	Cisplatin (100 mg/m^2 BSA)	120 min	41–43°C
Ambrogi et al. (10)	N = 13	Cisplatin (80 mg/m^2 BSA); doxorubicin (25 mg/m^2 BSA)	60 min	42.5°C
Maury et al. (11)	N = 19	Cisplatin (50 mg/m^2 BSA); mitomycin (25 mg/m^2 BSA)	90 min	42°C
Markowiak et al. (12)	N = 29	Cisplatin (100–175 mg/m^2 BSA); doxorubicin (0–65 mg)	60 min	42°C

Note: Body surface area (BSA) was calculated according to the Mosteller formula: BSA = 0.016667×Weight$^{0.5}$×Height$^{0.5}$

The objectives of this study are to collect data on the standard procedure of S-HITHOC at our center and to evaluate S-HITHOC in the perioperative outcomes, including the length of postoperative hospital stay, treatment-related adverse events and complications, and European Organization for Research and Treatment of Cancer Quality of Life Questionnaires (EORTC QLQ-C30) scores.

References

1. de Jong WK, Blaauwgeers JL, Schaapveld M, Timens W, Klinkenberg TJ, Groen HJ. Thymic epithelial tumours: a population-based study of the incidence, diagnostic procedures and therapy. Eur J Cancer. Jan 2008;44(1):123-30. doi:10.1016/j.ejca.2007.11.004

2. Nakahara K, Ohno K, Hashimoto J, et al. Thymoma: results with complete resection and adjuvant postoperative irradiation in 141 consecutive patients. J Thorac Cardiovasc Surg. Jun 1988;95(6):1041-7.

3. Turna A, Sarbay İ. Multimodality approach in treatment of thymic tumors. J Thorac Dis. Dec 2020;12(12):7626-7634. doi:10.21037/jtd-20-818

4. Hejna M, Haberl I, Raderer M. Nonsurgical management of malignant thymoma. Cancer. 1999/5/1/ 85(9):1871-84.

5. Refaely Y, Simansky DA, Paley M, Gottfried M, Yellin A. Resection and perfusion thermochemotherapy: a new approach for the tre atment of thymic malignancies with pleural spread. The Annals of thoracic surgery. 2001/8// 72(2):366-70. doi:10.1016/s0003-4975(01)02786-2

6. de Bree E, van Ruth S, Baas P, et al. Cytoreductive surgery and intraoperative hyperthermic intrathoracic ch emotherapy in patients with malignant pleural mesothelioma or pleural metastases of thymoma. Chest. 2002/2// 121(2):480-7. doi:10.1378/chest.121.2.480

7. Ried M, Potzger T, Braune N, et al. Cytoreductive surgery and hyperthermic intrathoracic chemotherapy perf usion for malignant pleural tumours: perioperative management and clin ical experience. European journal of cardio-thoracic surgery : official journal of the European Association for Cardio-thoracic Surgery. 2013/4// 43(4):801-7. doi:10.1093/ejcts/ezs418

8. Yellin A, Simansky DA, Ben-Avi R, et al. Resection and heated pleural chemoperfusion in patients with thymic epithelial malignant disease and pleural spread: a single-institution experience. J Thorac Cardiovasc Surg. Jan 2013;145(1):83-7; discussion 87-9. doi:10.1016/j.jtcvs.2012.10.013

9. Yu L, Jing Y, Ma S, Li F, Zhang Y-F. Cytoreductive surgery combined with hyperthermic intrapleural chemothe rapy to treat thymoma or thymic carcinoma with pleural dissemination. Onco Targets Ther. 2013/5/10/ 6:517-21. doi:10.2147/OTT.S41347

10. Ambrogi MC, Korasidis S, Lucchi M, et al. Pleural recurrence of thymoma: surgical resection

followed by hyperthermic intrathoracic perfusion chemotherapydagger. Eur J Cardiothorac Surg. Jan 2016;49(1):321-6. doi:10.1093/ejcts/ezv039

11. Maury JM, Girard N, Tabutin M, et al. Intra-Thoracic Chemo-Hyperthermia for pleural recurrence of thymoma. Lung Cancer. Jun 2017;108:1-6. doi:10.1016/j.lungcan.2017.02.014

12. Markowiak T, Neu R, Ansari MKA, et al. Surgical Cytoreduction and HITOC for Thymic Malignancies with Pleural Dissemination. The Thoracic and cardiovascular surgeon. 2021/3// 69(2):157-164. doi:10.1055/s-0039-1700883

2. Objectives and outcomes measures

The co-primary outcome measures in this trial are as follows:

- Treatment-related adverse events: All types and the severity of adverse events that are related to each patient's treatment will be recorded. Clavien-Dindo Classification will be used to grade the severity of the postoperative treatment-related complications. Treatment-related adverse events will be stratified according to the Common Terminology Criteria for Adverse Events, Version 5.0 (CTCAE v5.0).
- Length of postoperative hospital stay: This is defined as the duration from the date of surgery to the date that each patient meets the inclusion criteria for hospital discharge. The criteria for hospital discharge is no fever, normal chest X-ray, daily drainage less than 200 ml, and a good physical status.
- EORTC QLQ-C30 score for overall QoL: Patients will be evaluated 1 day before surgery and then postoperatively in the 1st, 3^{rd,} and 6th months using the EORTC QLQ-C30 (V3.0). QoL scores will be linearly converted to a scale ranging from 0 to 100 according to the EORTC guidelines (14). On this scale, a score of 100 represents the best QoL, while a score of zero indicates the worst QoL.

The secondary outcome measures in this trial are as follows:

- VAS scores for postoperative pain: Patients' cumulative daily pain score will be obtained at rest using the VAS from postoperative 0 to 72 hours. On the VAS, a score of zero indicates no pain, while a score of 100 mm represents the worst possible pain. The VAS has the following ranges for pain: painless (0–4 mm), mild pain (5–44 mm), moderate pain (45–74 mm), and severe pain (75–100 mm) (15).
- Postoperative drainage duration: This is defined as the duration from the day when the drainage tubes are placed until the day when the drainage tubes are moved.
- Postoperative drainage volume: This is defined as the total drainage volume during the hospital stay. Data on the daily drainage volume will be extracted from the care sheet, and the total amount of drainage will be calculated.
- PFS: This is defined as the duration from the date of S-HITHOC to the date of the first progression. Disease progression is defined as the new locoregional lesion (anterior mediastinal lesion, lung, pericardiac, or pleural invasion) or a new metastatic lesion (pericardial dissemination, pleural dissemination, or distant organ metastasis).
- OS: This is defined as the duration from the date of S-HITHOC to the date of death.

3. TRIAL DESIGN

3.1 Summary of Trial Design

A Prospective, Single-Arm, Phase II Study

3.2 Number of centers

Single center

3.3 Intervention and control

Intervention group: Cytoreductive Surgery and Hyperthermic Intrathoracic Chemotherapy

3.4 Sample Size

The sample size was based on estimates of major treatment related adverse event rates in patients who underwent S-HITOC. A major treatment related adverse events rate within 15% was

considered manageable and a rate greater than 30% was considered unsafe. Therefore, 37 patients were required at a $p = 0.1$ significance level with 80% power to detect a 15% difference in the major treatment related adverse events rate.

3.5 Participant identification

3.5.1 Trial Participants

The Investigator will determine trial participant eligibility based on the criteria given below.

3.5.2 Inclusion Criteria

The participant may enter the trial if **ALL** of the following apply:

- Puncture biopsy, thoracoscopic/mediastinal biopsy, or surgery to confirm pathologically thymic epithelial tumor (TETs)
- Imaging examination shows TETs with pleural spread or recurrence, and the mediastinal MDT team considers HITOC suitable.
- Patients with ≥16 and ≤80 years old.
- ASA I-II.
- The patients should have no functional disorders in the main organs.
- There was no history of other malignant carcinomas.
- The duration from the last chemotherapy was >4 weeks, the duration from the last radiotherapy was >6 weeks, and the duration from the last immunotherapy was >6 weeks.
- Not allergic to cisplatin or doxorubicin.
- The patients should be able to understand our research and sign the informed consent.

3.5.3 Exclusion Criteria

The participant may not enter the trial if **ANY** of the following apply:

- Imaging or pathological examination shows TETs without pleural spread or recurrence, or with pericardial dissemination or extrathoracic metastasis.
- Patients with lymphoid system, neurogenic or reproductive system carcinoma.
- Patients who have been receiving chemotherapy, radiotherapy, immunotherapy, or targeted

therapy.

- Patients with myasthenia gravis in unstable or acute exacerbation stage.
- The patients have been proven history of congestive heart failure, angina without good control with medicine; ECG-proved penetrating myocardial infarction; hypertension with bad control; valvulopathy with clinical significance; arrhythmia with high risk and out of control.
- The patients have the severe systematic intercurrent disease, such as active infection or poorly controlled diabetes; coagulation disorders; hemorrhagic tendency or under-treatment of thrombolysis or anticoagulant therapy.
- Female who is positive for a serum pregnancy test or during lactation period.
- The patients have a history of organ transplantation (including autologous bone marrow transplantation and peripheral stem cell transplantation.
- The patients have a history of peripheral nerve system disorders, obvious mental disorders, or central nerve system disorders.
- The patients attend other clinical trials.

3.5.4 Discontinuation/Withdrawal of Participants from Trial

Each participant has the right to withdraw from any aspect of the trial at any time.

3.5.5 Withdrawal from Follow Up

Participants may withdraw from the follow-up regime. If so, data up to the point of withdrawal will be collated and analyzed accordingly.

3.5.6 Withdrawal of Consent

If they do, this will be recorded and they can discuss treatment options with their surgeon.

4. Qualification of the responsible surgeons

The responsible surgeons should meet the following qualifications:

Completing at least 50 cases of thymectomy (including subxiphoid approach and lateral intercostal approach) respectively; also, Completing at least 20 cases of pleurotomy/decortication (P/D) and/or extended pleurectomy/decortication.

5. Patient Identification and Recruitment

Patients will be identified at outpatient clinics by participating surgeons and their clinical care team. Then a chest enhanced CT or MRI will be recommended for the potential patients to assess the mediastinal mass. Then the CT or MRI radiology imaging scan will be reviewed by a multidisciplinary team. If diagnosed clinically as “TETs with Pleural Spread or Recurrence”, the patients will be seen by the local research nurses to discuss the study further and the PI or designee will confirm eligibility.

Potentially eligible patients will be given an invitation letter and information sheet explaining why they have been approached. The research team will discuss the study details with the patient. If patients are willing to proceed into the study, the research nurse or clinician will ask the patient whether they agree to consent at that time or need up to a week to discuss with family or friends and agree on an arrangement to confirm their decision.

If a further appointment is necessary for the patient to return to give consent and complete the baseline assessment, all efforts will be made to ensure this appointment is as convenient for the patient as possible and may coincide with their pre-operative assessment appointment. The preoperative assessment appointments are routinely scheduled for a short time before the patient’s scheduled operation date.

Data related to the number of potential patients identified and approached will be recorded on screening logs. The screening log will also depict the number of patients who, once assessed, are deemed ineligible and also those who decline to participate. Reasons for eligible patients not participating will be requested, but do not have to be given. Screening logs will not contain patient identifiable information.

5.1 Baseline Assessments

Prior to randomization the following information will be collected:

(1) Patient demographics (gender, age at surgery, body mass index);

(2) Tumor characteristics (diameter, location, Masaoka-Koga stage);

(3) Pre-operative examination results (FEV_1, FVC);

(4) Summary medical history;

(5) Summary medication history (current medication and duration of current medication, changes to current medication in the last 6 months, any history of prior opiate use);
(6) Pain Visual Analogue Scale (0-10) at rest;
(7) EORTC QLQ-C30 score;
(8) Patient expectations of treatment.

5.2 Eligibility Consulting

Contact Information and Working Hours of Research Committee:

Add: Research Committee of Esophageal Cancer Treatment, Zhongshan Hospital, Fudan University

Tel: 021-64041990-2917

Working Hours: Monday to Friday, 9:00 to 17:00 (except weekends and holidays)

Contact Information:

Prof. Jianyong Ding

Add: Department of Thoracic Surgery, Zhongshan Hospital, Fudan University, Fenglin Road 180, Shanghai 200032, China.

Phone Number: 086-18616881268

Email: ding.jianyong@zs-hospital.sh.cn

5.3 Informed Consent

Informed consent will be taken by a medically-qualified and suitably experienced investigator. All delegates must be authorised by the Chief/Principal Investigator to obtain consent. The Investigator is responsible for ensuring that the trial consent procedures comply with current applicable GCP Regulatory and ethical requirements. The Investigator must be satisfied that the patient has made an informed decision before taking consent. The participant must personally sign and date the latest approved version of the Informed Consent Form before any trial specific procedures are performed. Consent will be verbally re-confirmed on the day of

surgery.

Written and verbal versions of the Participant Information and Informed Consent will be presented to the participants detailing no less than: the exact nature of the trial; what it will involve for the participant; the implications and constraints of the protocol; the known side effects and any risks involved in taking part. It will be clearly stated that the participant is free to withdraw from the trial at any time for any reason without prejudice to future care, and with no obligation to give the reason for withdrawal.

The participant will be allowed as much time as wished to consider the information, and the opportunity to question the Investigator, their GP or other independent parties to decide whether they will participate in the trial. The patient and the Investigator must personally sign and date the current approved version of the informed consent form in each other's presence. A copy of the signed Informed Consent will be given to the participant. The original signed form will be retained at the trial site.

6. Treatment

Surgery technique

The date for surgery is defined as Day 0. Patients will be monitored by electrocardiogram, arterial catheter, pulse oximeter, end-tidal carbon dioxide, and urine output. Minimally invasive (if possible) or open surgery will be performed on all enrolled participants to reduce the tumor burden. During the surgery, the surgeons will try to remove all the visible tumor lesions as completely as possible and will choose the most appropriate surgical method based on the location and number of the lesions. Partial pleurectomy will be used for oligometastatic pleural nodules. Complete pleurectomy will be performed selectively, taking into account the safety of the operation and will always be used for multiple nodules involving extensive dissemination on the parietal pleura. Partial diaphragm resection combined with diaphragm repair will be used for diaphragm dissemination. Partial pericardial resection combined with pericardiac repair will be applied to any tumor lesions that have spread into the pericardium. Two 28F drainage tubes will be placed in the pleural space; one will be located at the seventh intercostal space in the middle axillary line for outflow drainage,

and the other will be located at the sixth intercostal space in the anterior axillary line for inflow. During the operation, the patients will receive generous fluids and blood transfusion (if necessary) to maintain adequate blood pressure and urine output. The patients will then be returned to their ward or the intensive care unit (ICU) after the operation. Patient controlled analgesia will be used for postoperative analgesia.

Complete resection (R0) and perioperative complications will be recorded in the case report forms (CRFs). R0 is defined as the microscopic removal of all gross tumors/lesions. Subtotal resection is defined as microscopic minimal residual disease. Partial resection is defined as macroscopic incomplete resection. Perioperative complications might include arrhythmia, respiratory distress, and hemorrhage.

HITHOC technique

The safety of HITHOC for each patient will be assessed by the mediastinal MDT team before HITHOC. The patients enrolled in the study will be given HITHOC regardless of their resection status. HITHOC will be performed at the ward, ICU, or surgical rooms on Days 1 and 2 if the patients are without fever, hemorrhage, atelectasis, or massive pleural effusion. The HITHOC team comprises one chief surgeon, one technician, and one nurse. When performing HITHOC, we will use the BR-TRG-I type device (Guangzhou Bright Medical Technology, Guangzhou, China), a dedicated perfusion system approved by the Chinese Food and Drug Agency. The BR-TRG-I body cavity thermal perfusion therapy system consists of four parts: a control system, an external circulation system, a heat exchanger, and an internal circulation system (Figure S1–2). The BR-TRG-I device is equipped with a dedicated heat exchanger to ensure a high temperature for the locoregional oncological treatment. The extra-corporal circuit will be primed with lactated ringer solution. Fluids will flow through the outflow drain until all the air is removed from the pleural space through a sidearm in the outflow pipe, and the initial heating perfusion will then begin. Circulation flows of 400 to 600 mL/min with an inflow temperature of 42–43°C will be required to maintain the desired temperature. Doxorubicin will be infused at 25mg/m2 and perfused on Day 1. Cisplatin will be infused at 50mg/m2 and perfused on Day 2. The inflow chest tube will be removed

and the excess pleural fluid will be allowed to flow freely through the outflow chest tube to a collecting system after HITHOC is performed for approximately 60 minutes.

7. Postoperative Management

All the adverse events and postoperative complications will be recorded, assessed, and treated. The Visual Analog Scale (VAS) score will be recorded to evaluate postoperative pain before the operation, and 1 and 3 days after the operation. We will use the EORTC QLQ-C30 (V3.0) to assess the quality of life (QoL) of the patients at 30, 90 and 180 days after surgery. For all patients who receive S-HITHOC, chest computed tomography scans will be performed every 3 months for the first postoperative 6 months, then, every 6 months for the first two years, and finally, annually for the rest of their lives. Further examinations will be administered as necessary, including ultrasound, puncture biopsy, and positron emission tomography–computed tomography scans.

8. Data Management

8.1 Case Report Form (CRF)

8.1.1 Types and Submission Deadline

CRF used in this study and the submission deadline is as follows:

Case screening: 7 days prior to surgery (time frame: 3 days)
Enrolling: submitted to the data center one day prior to surgery
Surgery: within 1 day after surgery
Postoperation-Discharge: within 3 days after the first discharge
Follow-up records: 7 days after each follow-up point

8.1.2 Transmission Method

Paper CRF and web-based eCRF form are used for data submission.

8.1.3 Amendment

After the start of the study, if the CRF is found lack of necessary data items or unclear items, under the premises of ensuring the amendment of the CRF does not cause medical and economic burden and increased risks to the selected patients, the CRF can be modified after the Research Committee adopt it through discussing at the meeting. If the amendment of the CRF does not

require to modify the study protocol, this study protocol will not be modified. That whether it is necessary to submit a report or lodge an application to each research center's IRB for the CRF amendment should follow the provisions of various centers.

8.2 Monitoring and Supervision

In order to study whether the implementation follows the protocol safely, to study whether to collect the data correctly, monthly monitoring should be implemented during the period of selection of cases in principle. The monitoring is based on the hospital visit to compare the difference between and the original data and data submitted.

The periodic data report completed by the data center should be submitted to the Research Committee, the Research Responsible Person and Efficacy and Safety Evaluation Committee, and should be discussed and analyzed in accordance with relevant monitoring provisions. The regular monitoring is to aim at feedback, improving the scientific, ethical nature of the study rather than trying to expose study or hospital issues. The Research Committee, the Research Responsible Person, and the person in charge of research participating hospitals should strive to improve and to avoid the problems pointed out in the regular monitoring reports.

8.2.1 Monitoring Items

Data collection completed status: Selected registration number (cumulative/different time of period, all hospitals/different hospitals);
Eligibility: Ineligible patients/potentially ineligible patients (different hospitals);<br>Different end of treatment, the reasons for suspension/end (different hospitals) in the study protocol;
Background factors, pre-treatment report factors, post-treatment report factors when selected for registration;
Severe adverse events (different hospitals)
Adverse events/adverse reactions (different hospitals)
Proportion of conversion to open surgery (different hospitals)
Protocol deviation (different hospitals)
Progress and safety of the study, other issues

8.2.2 Acceptable Range of Adverse Events

Based on the qualification of the research centers in this study, in general, treatment-related death and life-threatening complications caused by surgeries do not happen basically; the percent of more than 2% is considered unacceptable. If treatment-related death is suspected having a causal relationship with the surgery is determined, adverse events on each patient should be respectively reported to the Efficacy and Safety Evaluation Committee. If the number of treatment-related deaths having a causal relationship with the surgery is up to 3, the final incidence proportion of adverse events will be apparently more than 2%, and therefore the inclusion of patients must be immediately suspended. Whether the study can continue to proceed should be determined until reviewed by the PI Efficacy and Safety Evaluation Committee.

8.2.3 Deviation/Violation of Study Protocol

Surgical resection, clinical examinations, or toxicity, efficacy evaluation and so on failing to be conducted in accordance with the study protocol are the deviation of the study protocol. When the monitoring is carried out, deviations developed by the Data Center and Research Committee in advance (allowed to after the start of the study in special circumstances) beyond the acceptable range specified in each study center should be included in the monitoring report in the form of "cases of deviation possibility", and divided into any arbitrary one of the following after discussed by the Research Committee.

8.2.3.1 Violation

Clinically inappropriate, a deviation at least complying with one of the following items specified in the protocol is called "violation":

(1) Affecting the study endpoint evaluation;

(2) The responsibility lays the doctor in charge/hospital;

(3) Intentional or systematic

(4) Significant danger or the degree of deviation

(5) Papers should record content violation in principle.

8.2.3.2 Acceptable deviation

The acceptable deviation represents the acceptable range of each item set by the Research

Representative/Committee and the data center before or after the beginning of the study. If it is within an acceptable range of deviation set in advance, no record is required in the monitoring report.

8.2.3.3 Deviation

Items that do not comply with 18.2.3.1 or with 18.2.3.2 are deviation items.

Specific deviations that occur several times should be recorded as much as possible when the paper is published.

When the monitoring report is discussed, the deviation should be classified as the following:

(1) Deviated from undesired results: should be reduced;

(2) Deviation (inevitable): not to be actively reduced;

(3) Deviation (clinically appropriate): positive affirmation of the judgment by the doctor in charge/ hospital.

9. Provisions on Adverse Events

The evaluation in this study refers to CTCAE v5.0 and "Accordion Severity Grading System".

9.1 Evaluation

Evaluation of adverse event/adverse reaction comprehensively refers to the [CTCAE v5.0].

CTCAE v5.0, the so-called "Adverse Event", "all observed, unexpected bad signs, symptoms and diseases(abnormal value of clinical examination are also included) in the treatment or disposal, regardless of a causal relationship with the treatment or intervention. So it can be divided into two types based on whether there is a causal relationship or not. Therefore, even if events that "obviously caused by primary disease (cancer)" or caused by supportive therapy or combination therapy rather than the study regimen treatment (protocol treatment) are defined as "adverse events".

For adverse event data collection strategy, the following principle should be complied with in this study: Adverse events within 30 days from the last treatment day of the study regimen or hospitalization before first discharge (postoperative hospital stay > 30 days) (protocol treatment) should be collected entirely, regardless of the presence or absence of a causal relationship. (When

adverse events are reported, the causality and classification of adverse events are separately discussed)

9.2 Reporting

When "severe adverse events" or "unexpected adverse events" occur, the Research Responsible Person of each research center should report to the Research Committee/PI (Lijie Tan). Before the start of the study, the Research Committee should send the report template to each research center in advance. When "severe adverse events" or "unexpected adverse events" occur, the Research Responsible Person of each research center should report them to the Research Committee/PI (Lijie Tan).

Adverse events based on the relevant laws and regulations should be reported to the province (city) Health Authority at the location of each research center. Severe adverse events based on clinical research-related ethical guidelines should be reported to the person in overall charge of the medical institution. The appropriate reporting procedures should be completed in accordance with the relevant provisions of all medical institutions at the same time. The person in charge of each center should hold obligations and responsibility for the emergency treatment of patients with any degree of adverse events to ensure patient safety.

9.2.1 Adverse Events with Reporting Obligations

9.2.1.1 Adverse Events with Emergency Reporting Obligations

Any of the following adverse events is the object that any adverse event should be reported urgently to: All patients died during the course of treatment or within 30 days from the last treatment day, regardless of the presence or absence of a causal relationship with the study regimen treatment. If cases are withdrawn of treatment, even if the latter treatment has begun, those patients also belong to emergent reporting objects, as long as within 30 days from the last treatment day or during hospitalization (hospital stay > 30 days). (day 0 is the final treatment day and 30 days is starting from the next day); Those patients having a causality with the treatment (any of definite, probable, possible) are also emergent reporting objects.

9.2.1.2 Adverse Events with Regular Reporting Obligations

Any one of the following adverse events is a regular reporting object:

(1) After 31 days from the last treatment day, death that cannot rule out the causal relationship with treatments, including suspected treatment-related death; death due to apparent primary disease is excluded.

(2) Unexpected Grade 3 adverse events: Grade 3 adverse events are not recorded in the expected adverse events.

(3) Other significant medical events: adverse events that the study group deems are found to bring essential and potentially permanent, significant impact on their offspring (except for MDS myelodysplastic syndrome, and secondary cancer).

(4) Adverse events among above (2)-(4), determined to have a causal relationship (any of definite, probable, and possible) with the study regime are regular reporting objects.

9.2.2 Reporting Procedure

9.2.2.1 Emergency Reporting

When emergent adverse events of emergency study reporting objects happened, the doctor in charge will quickly report it to the Research Responsible Person of the research participating hospitals. Where no contact can be gotten with the Research Responsible Person of the hospital, the coordinator, or the doctor in charge of the hospital must perform the responsibility instead.

First Reporting: Within 72 hours after the occurrence of adverse events, the Research Responsible Person of the hospital should complete the "AE/AR/ADR first emergency report" and send it to the Research Committee by FAX and telephone.

Second Reporting: The Research Responsible Person of each research participating hospital completes the "AE/AR/ADR Report" and a more detailed case information report (A4 format), and then fax the two reports to the Research Committee within 15 days after the occurrence of adverse events. If any autopsy examination, the autopsy result report should be submitted to the Research Committee.

9.2.2.2 General Reports

The Research Responsible Person of each research participating hospital completes the "AE/AR/ADR report", and then fax it to the Research Committee within 15 days after the occurrence of adverse events.

9.3 Responsibilities and Obligations

9.3.1 Judgment of Study Discontinuation and Necessity for Sending an Emergency Notice to the Hospital

After the receipt of the report of the Research Responsible Person of the research participating hospital, the Research Committee reply to the Research Responsible Person of the unit for confirmation and negotiation, and then they jointly determine the urgency, importance, and influence of reporting events; if necessary, they temporarily stop the study, and contact with all research participating hospitals to take emergency notification countermeasures. According to the severity of urgency, data center and research participating hospitals can be contacted by telephone or instrument FAX as soon as possible after the initial contact by phone.

9.3.2 Report to PI Efficacy and Safety Evaluation Committee

Adverse events with reporting obligations in the emergency reports or regular reports to the Research Responsible Person of research participating units, the Research Committee should submit a report to the Efficacy and Safety Evaluation Committee within 3 days after the occurrence of adverse events and request a review that whether the reason analysis of and solution to the adverse events by the Research Responsible Person are appropriate.

At that time, “AE/AR/ADR First Emergency Report” and “AE/AR/ADR Report” submitted by the research participating hospital should include the discussion results and countermeasures of the Research Committee/Research Responsible Person(including the judgment of research continue/discontinue). For death within 30 days, treatment-related death among death after 31 days, not only the course of individual patient are included, but also consideration given to that whether the frequency of occurrence falls within the expected range are included. If the frequency of occurrence exceeds the expected range, it should be faithfully recorded in the “II classification of adverse events-others” of “AE/AR/ADR Report”.

9.3.3 Notice to the Research Participating Hospitals

After submitting the report to the CLASS Efficacy and Safety Evaluation Committee, the Research Committee/Research Responsible Person should notify the efficacy, and review, proposal content of the Efficacy and Safety Evaluation Committee in written form to all research

participating hospitals.

If failing to submit the report to the Efficacy and Safety Evaluation Committee, the Research Committee/Research Responsible Person should report their judgment in written form to the Research Responsible Person of a research participating hospital that submitted the report.

9.3.4 Discussion of Adverse Events under Regularly Monitoring

During the regular monitoring, the Research Committee/Research Responsible Person should carefully discuss, study adverse events in the monitoring report submitted by the research data center to confirm no missing report by each research participating hospital. The existence or inexistence of under-reporting adverse events should be clearly documented in the discussion results of [regularly monitoring report] of the Research Committee.

9.4 Review of Efficacy and Safety Evaluation Committee

The Efficacy and Safety Evaluation Committee reviews and discusses the report in accordance with the procedures recorded in the Clinical Safety Information Management Guideline, and raises the recommendations in written form for the Research Responsible Person, including whether to continue to enroll the study objects or whether to need to modify the study protocol.

10. Ethics

10.1 Responsibilities of Investigators

The investigators are responsible for the implementation of this study in its center. The investigators will ensure the implementation of this study in accordance with the study protocol and in compliance with the Declaration of Helsinki, as well as domestic and international ethical guiding principles and applicable regulatory requirements. It is especially noted that the investigators must ensure that subjects giving the written informed consent can be enrolled in this study only.

10.2 Information and Informed Consent of Subjects

An unconditional prerequisite for subjects to participate in this study is his/her written informed consent. The written informed consent of subjects participating in this study must be given before study-related activities are conducted.

Therefore, before obtaining informed consent, the investigators must provide sufficient information to the subjects. In order to obtain informed consent, the investigators will provide the information page of subjects, and the information required to comply with the applicable regulatory requirements. While providing written information, the investigators will orally inform the subjects of all the relevant circumstances of this study. In this process, the words used must be fully, easily understood by non-professionals, so that they can sign on the informed consent form according to their willingness based on subjects' fully understanding of this study.

The informed consent form must be signed and dated personally by the subjects and investigators. All subjects will be asked to sign on the informed consent form to prove that they agree to participate in the study. The signed informed consent form with signature and date should be kept in the research center where the investigators are located and must be properly safe kept for the future review at any time during the audit, inspection, inspection period. Before participating in the study, the subjects should provide a copy of signed and dated informed consent form.

At any time, as long as access to important new information that may be related to the consent of the subjects, the investigators will revise the information pages and any other written information provided to the subjects and re-submit them to the IEC/IRB for review and raising a favorable opinion. The revised information agreed will be provided to each subject participating in the study. The researchers will explain the changes made to the previous version of ICF to the subjects.

10.3 Identity and Privacy of Subjects

After obtaining an informed consent form, each selected subject is assigned with subject number (Allocation Number, AN). This number will represent the identity of the subject in the whole study and the clinical research database for the study. The collected data of subjects in the study will be stored in the ID. In the entire study, various safety measures to minimize leaking risks in the utilization process of personal information will be taken, including: (1) only the investigators are able to link the research data of the subjects with themselves through the identify table kept in the research center after authorized; (2) in the raw data auditing on-site conducted by the supervisors of this study, as well as relevant inspection and inspection visit by the supervision departments, the personnel engaging above activities may view the original medical information of

subjects that will be kept strictly confidential. Data collection, transmission, handling, and storage of subjects will comply with the data protection and privacy regulations. This corresponding information will be provided to the subjects, and the subjects are asked to provide their consent for the treatment procedures of above data in accordance with national regulations.

10.4 Independent Ethics Committee or Institutional Review Committee

Before beginning the study, the Research Center will be responsible for submitting the study protocol and relevant documents (informed consent form, subject information page, CRF, and other documents that may be required) to the Independent Ethics Committee (IEC)/ Institutional Review Board (IRB) to obtain their favorable opinion/approval. The favorable opinion/approval documents of the IEC/IRB will be archived in the research center folders of the investigators. Before obtaining the written proof of favorable opinions/approval of the IEC/IRB, the investigators are forbidden to begin the study in the center. The IEC/IRB will be asked to provide the written proof of the date of the favorable opinions/approval meeting and the written proof of the members presenting at the meeting and voting members. The IEC/IRB should provide the written proof of the favorable opinion/approval, recording the reviewed study, protocol version, and Informed Consent Form version. If possible, a copy of the minutes should also be obtained. In the case of major revisions in this study, the amendment of the study protocol will be submitted to the IEC/ IRB prior to performing. In the course of the study, the relevant safety information will be submitted to the IEC/IRB in accordance with national regulations and requirements.

10.5 Supervisory Authority

The study protocol and any relevant documents (for example, the study protocol, the subject's informed consent form) will be submitted according to the Ethical Review Approach of Biomedical Research Involving Human Beings (Trial) (2007) and the applicable regulatory requirements of our country or will notify the ethical review guidance counseling organization of the provincial health administrative departments at the location of each research center.

11. Organizations and Responsibilities of Study

11.1 Research Committee

Being responsible for developing study protocol, auditing eligibility for inclusion, and guiding

the interpretation of informed consent; being responsible for the collection of hazardous/adverse event reports, guiding the clinical diagnosis and treatment of such events, and the emergency intervention of serious adverse events.

11.2 Data and Safety Monitoring Board

DSMB is responsible for the supervision of efficacy, the safety of this study, supervising of all aspects performed of the study, and licensing before the release of the validity of the study results.

Person in Charge of DSMB: CRO

11.3 Independent Ethics Committee/Institutional Review Board (IEC/IRB)

Being Responsible for evaluating this study in order to determine "whether to minimize risks that the subjects are exposed to" and "whether the risks that the subjects are exposed to are reasonable compared to expected benefits". The independent Ethics Committee/Institutional Review Board (IEC/IRB) at the location of each research center is responsible for the ethics review of all research participating units.

12. Publications of Research Results

The publication of the research results of the paper should follow the established principle of the publication period in the study protocol. When there are no definite established policies of the research group, the publication of the paper should follow the following principle: the main statistical analysis, the final statistical analysis, and the final complete public paper written contributions to journals in English. Unless clearly provided in the study protocol, the methods of used statistical analysis and the final statistical analysis cannot be published without approval of the Efficacy and Safety Evaluation Committee. However, excluding the results of the final statistical analysis of this study, the research representative or the Research Committee can publish the Society Paper (Abstract) to introduce of this study just need to obtain consent from the person in charge of the data center.

In principle, the author of the main published paper of the research results is firstly the Research Committee, followed by the research representative, the person in charge of statistics of the data center (the person in charge of statistical analysis for publication). The rest should follow

the paper written contribution rules. In order of the selected registration size of samples, the Research Responsible Persons of all research centers are listed as co-authors. All co-authors shall review the paper and agree to publish it before the paper submission. If the consent cannot be gotten from an investigator because of disagreeing with the published content, the research representative has the right not to list the investigator as co-author.

For the overall data collected in this study, if any person in charge of research center need make a secondary analysis or make an analysis for other research purposes, the consent of the Research Committee shall be gotten; when a person in charge of research center need to use the data of his group to make the speech on the academic conference, the data source should be noted and informed the Research Committee.

The publication of the primary objectives should be penned by people in charge of the research, principally. The publication of the second objectives or secondary analysis for the results can be negotiated by the person in charge of research participating units of this research organization but must obtain the permission of the person in charge of the whole research.

The person in charge of the research center has right to save their single-center data but should follow the privacy principles; For the results, form, the content of published single-center data, the relevant responsibilities should be at their own risk. The Research Committee does not assume any responsibility; the use of single-center data must be informed and obtain the recognized accuracy from the CLASS data center; the single-center data of statistical analysis must be marked to derive from this study of the CLASS in order to avoid repeat inclusion at the time of systemic analysis.

Without the approval of both the Research Committee and the data center, No Research Committee personnel cannot directly obtain the overall data and results of statistical analysis of this study from the data center.
