# Supplementary material for: Cytoreductive surgery and hyperthermic intrathoracic chemotherapy in thymic epithelial tumors with pleural spread or recurrence: a prospective, single-arm, phase II study
Source: Nat Commun. 2025 Jun 4;16:5175. doi: 10.1038/s41467-025-60386-0 (PMC12137697; doi:10.1038/s41467-025-60386-0)
Supplement: Supplementary file 2 — Reporting Summary [file 41467_2025_60386_MOESM2_ESM.pdf]

nature portfolio

Corresponding author(s): Lijie Tan, Jianyong Ding

Last updated by author(s): Apr 27, 2025

Reporting Summary

Nature Portfolio wishes to improve the reproducibility of the work that we publish. This form provides structure for consistency and transparency in reporting. For further information on Nature Portfolio policies, see our Editorial Policies and the Editorial Policy Checklist.

Statistics

For all statistical analyses, confirm that the following items are present in the figure legend, table legend, main text, or Methods section.

n/a	Confirmed	
☐	☒	The exact sample size (n) for each experimental group/condition, given as a discrete number and unit of measurement
☐	☒	A statement on whether measurements were taken from distinct samples or whether the same sample was measured repeatedly
☐	☒	The statistical test(s) used AND whether they are one- or two-sided<br>*Only common tests should be described solely by name; describe more complex techniques in the Methods section.*
☐	☒	A description of all covariates tested
☐	☒	A description of any assumptions or corrections, such as tests of normality and adjustment for multiple comparisons
☐	☒	A full description of the statistical parameters including central tendency (e.g. means) or other basic estimates (e.g. regression coefficient) AND variation (e.g. standard deviation) or associated estimates of uncertainty (e.g. confidence intervals)
☐	☒	For null hypothesis testing, the test statistic (e.g. F, t, r) with confidence intervals, effect sizes, degrees of freedom and P value noted<br>*Give P values as exact values whenever suitable.*
☒	☐	For Bayesian analysis, information on the choice of priors and Markov chain Monte Carlo settings
☐	☒	For hierarchical and complex designs, identification of the appropriate level for tests and full reporting of outcomes
☒	☐	Estimates of effect sizes (e.g. Cohen's d, Pearson's r), indicating how they were calculated

Our web collection on statistics for biologists contains articles on many of the points above.

Software and code

Policy information about availability of computer code

Data collection	The beginning of follow-up was defined as the date of cytoreductive surgery regardless of whether preoperative treatment was used. The Mann-Whitney test was used to compare the EORTC QLQ-C30 QoL scores and VAS scores at baseline and after treatment, and the Student's t-test was used to compare glucocorticoid usages. Progression was defined as documented intrathoracic and/or extrathoracic, ipsilateral and/or contralateral tumor detection by cytology/histology and/or imaging. PFS was defined as the time from surgery to the first objective tumor progression or death from any cause, whichever occurred first. OS was defined as the time from surgery to death due to any cause. The cut-off date for the last follow-up was June 30, 2024. PFS and OS were calculated using the Kaplan-Meier method with the log-rank test. The Cox proportional hazards regression model was used to explore the potential risk factors of PFS, and variables with p values <0.25 in the univariate analysis were entered into the multivariable analysis.
Data analysis	R software (version 4.3.1) was used to perform statistical analyses, and the ROC curve was used to calculate the best cutoff value of the PTI for predicting PFS.

For manuscripts utilizing custom algorithms or software that are central to the research but not yet described in published literature, software must be made available to editors and reviewers. We strongly encourage code deposition in a community repository (e.g. GitHub). See the Nature Portfolio guidelines for submitting code & software for further information.

Data

Policy information about availability of data

All manuscripts must include a data availability statement. This statement should provide the following information, where applicable:

- Accession codes, unique identifiers, or web links for publicly available datasets
- A description of any restrictions on data availability
- For clinical datasets or third party data, please ensure that the statement adheres to our policy

Source data are provided as a Source Data file. The data generated in this study have been deposited in the Figshare repository database without accession code. They can be freely and enduringly accessed (Figshare private link: https://figshare.com/s/c146c8764027700ee69a).

Research involving human participants, their data, or biological material

Policy information about studies with human participants or human data. See also policy information about sex, gender (identity/presentation), and sexual orientation and race, ethnicity and racism.

Reporting on sex and gender	In this study, we utilized self-reported sex as a basis for our analysis. The selection of participants was made without regard to sex or gender, with the primary aim of maximizing the sample size. It is important to note that sex was not analyzed in separate groups, nor was it taken into consideration during the statistical testing process. Within the cohort, 51.1% of the participants identified themselves as female.
Reporting on race, ethnicity, or other socially relevant groupings	No information on social groupings was used in this study. Eligible patients were adults with TETs confirmed by pathological examination and pleural spread or recurrence diagnosed by imaging, regardless of ethnicity. The patients in this study were all Chinese Han population, which has limited ethnic diversity. Therefore, our findings may not be generalizable to other settings and ethnic groups.
Population characteristics	All of 45 patients received S-HITOC are Chinese Han population. The participant characteristics are shown in Table 1. Among the patients, 22 (48.9%) were male, and seven (15.6%) were older than 65 years. Nineteen (42.2%) patients had comorbidities, and seven (15.6%) patients had a Charlson Comorbidity Index (CCI) of > 3. Pretreatment myasthenia gravis (MG) was observed in 12 (26.7%) patients, , of which 6 (13.3%), 4 (8.9%), 1 (2.2%), and 1 (2.2%) were classified as the Myasthenia Gravis Foundation of America (MGFA) class I, II, III, and IV, respectively.
Recruitment	Eligible patients were adults with TETs confirmed by pathological examination and pleural spread or recurrence diagnosed by imaging, regardless of ethnicity. Patients were excluded if they had an acute exacerbation of myasthenia gravis, renal dysfunction, a performance status score of more than 2, other malignant carcinomas, allergy to cisplatin or doxorubicin, or refused to participate in the study.
Ethics oversight	This research study complied with all relevant ethical regulations. The trial was conducted in accordance with the criteria set by the Declaration of Helsinki. The trial was approved by the Zhongshan Hospital Research Ethics Committee (ID: B2021-703R). This trial was registered at ClinicalTrials.gov (identifier: NCT05446935). All the investigators provided informed consent from each participant. This study was an open, single-arm, phase II trial that aimed to evaluate the perioperative safety and efficacy of S-HITOC in the treatment of TETs with pleural spread or recurrence. The trial protocol has been published(doi: 10.21037/jtd-23-759).

Note that full information on the approval of the study protocol must also be provided in the manuscript.

Field-specific reporting

Please select the one below that is the best fit for your research. If you are not sure, read the appropriate sections before making your selection.

☒ Life sciences ☐ Behavioural & social sciences ☐ Ecological, evolutionary & environmental sciences

For a reference copy of the document with all sections, see nature.com/documents/nr-reporting-summary-flat.pdf

Life sciences study design

All studies must disclose on these points even when the disclosure is negative.

Sample size	The sample size was based on estimates of major treatment related adverse event rates in patients who underwent S-HITOC. A major treatment related adverse events rate within 15% was considered manageable and a rate greater than 30% was considered unsafe. Therefore, 37 patients were required at a p = 0.1 significance level with 80% power to detect a 15% difference in the major treatment related adverse events rate. The experiments were not randomized. The investigators were not blinded to allocation during experiments and outcome assessment.
Data exclusions	Eligible patients were adults with TETs confirmed by pathological examination and pleural spread or recurrence diagnosed by imaging. Patients were excluded if they had acute exacerbation of myasthenia gravis, renal dysfunction, a performance status score of more than 2, other malignant carcinomas, allergy to cisplatin or doxorubicin, or refused to participate in the study. Primarily, 53 patients were registered. We exclude 2 patients confirmed mesothelioma, 2 patients withdraw, 1 patient had liver metastasis, 2 patients due had other tumors, and 1

	patient refused.
Replication	This study was an open, single-arm, phase II trial that aimed to evaluate the perioperative safety and efficacy of S-HITOC in the treatment of TETs with pleural spread or recurrence. The trial protocol has been published(doi: 10.21037/jtd-23-759). The experiments were not randomized. The investigators were not blinded to allocation during experiments and outcome assessment.
Randomization	This study was an open, single-arm, phase II trial. The experiments were not randomized. The Cox proportional hazards regression model was used to explore the potential risk factors of PFS, and variables with p values <0.25 in the univariate analysis were entered into the multivariable analysis.
Blinding	This study was an open, single-arm, phase II trial. The investigators were not blinded to allocation during experiments and outcome assessment.

Reporting for specific materials, systems and methods

We require information from authors about some types of materials, experimental systems and methods used in many studies. Here, indicate whether each material, system or method listed is relevant to your study. If you are not sure if a list item applies to your research, read the appropriate section before selecting a response.

Materials & experimental systems

n/a	Involved in the study
☒	☐ Antibodies
☒	☐ Eukaryotic cell lines
☒	☐ Palaeontology and archaeology
☒	☐ Animals and other organisms
☐	☒ Clinical data
☒	☐ Dual use research of concern
☒	☐ Plants

Methods

n/a	Involved in the study
☒	☐ ChIP-seq
☒	☐ Flow cytometry
☒	☐ MRI-based neuroimaging

Clinical data

Policy information about clinical studies
All manuscripts should comply with the ICMJE guidelines for publication of clinical research and a completed CONSORT checklist must be included with all submissions.

Clinical trial registration	NCT05446935
Study protocol	Yang X, et al. Cytoreductive surgery combined with hyperthermic intrathoracic chemotherapy for the treatment of thymic epithelial malignancies with pleural spread or recurrence (CHOICE): a study protocol for a prospective, open, single-arm study. J Thorac Dis.16,760-767 (2024).
Data collection	Between August 1, 2021, and February 29, 2024, 45 patients who received S-HITOC at Zhongshan Hospital of Fudan University were included in this work. The cut-off date for the last follow-up was June 30, 2024. All adverse events and postoperative complications were recorded and treated. VAS scores were recorded to evaluate postoperative pain before the operation and at 1 and 3 days after treatment. The QoL was evaluated at baseline and after treatment on the 1st, 30th and 60th days by using the European Organization for Research and Treatment of Cancer Quality of Life Questionnaire C-30 Scale (EORTC QLQ-C30) (V3.0). MG was classified according to the MGFA clinical classification system. The prognosis was evaluated according to the MGFA post-intervention status, and overall remission included CSR, PR, and MM. For all patients who received S-HITOC, chest computed tomography scans were performed every 3 months for the first 6 months after treatment, then every 6 months for the first two years and finally annually throughout their lifetime. Further examinations, including ultrasound, puncture biopsy, and positron emission tomography-computed tomography, were performed when needed.
Outcomes	The primary endpoint was a major treatment-related complication, defined as grade ≥3, according to the Clavien–Dindo classification (5th edition) and the Common Terminology Criteria for Adverse Events, Version 5.0 (CTCAE v5.0). The secondary outcomes included the LOS, QoL score, VAS score, PFS, and OS. VAS scores were recorded to evaluate pain by making a handwritten mark on a 10-cm line (VAS ruler) before the operation and at 1 and 3 days after treatment. The QoL was evaluated at baseline and after treatment on the 1st, 30th and 60th days by using the European Organization for Research and Treatment of Cancer Quality of Life Questionnaire C-30 Scale (EORTC QLQ-C30) (V3.0). MG was classified according to the MGFA clinical classification system.

Plants

Seed stocks	n/a
Novel plant genotypes	n/a
Authentication	n/a
